# Inhibition of Gamma-Secretase Promotes Axon Regeneration After a Complete Spinal Cord Injury

**DOI:** 10.3389/fcell.2020.00173

**Published:** 2020-03-20

**Authors:** Daniel Sobrido-Cameán, Diego Robledo, Daniel Romaus-Sanjurjo, Vanessa Pérez-Cedrón, Laura Sánchez, María Celina Rodicio, Antón Barreiro-Iglesias

**Affiliations:** ^1^Department of Functional Biology, CIBUS, Faculty of Biology, Universidade de Santiago de Compostela, Santiago de Compostela, Spain; ^2^The Roslin Institute and Royal (Dick) School of Veterinary Studies, The University of Edinburgh, Edinburgh, United Kingdom; ^3^Department of Genetics, Universidade de Santiago de Compostela, Santiago de Compostela, Spain

**Keywords:** notch, gamma-secretase, axon regeneration, GABA, baclofen, RNA-Seq, lamprey, spinal cord injury

## Abstract

In a recent study, we showed that GABA and baclofen (a GABAB receptor agonist) inhibit caspase activation and promote axon regeneration in descending neurons of the sea lamprey brainstem after a complete spinal cord injury (Romaus-Sanjurjo et al., 2018a). Now, we repeated these treatments and performed 2 independent Illumina RNA-Sequencing studies in the brainstems of control and GABA or baclofen treated animals. GABA treated larval sea lampreys with their controls were analyzed 29 days after a complete spinal cord injury and baclofen treated larvae with their controls 9 days after the injury. One of the most significantly downregulated genes after both treatments was a HES gene (*HESB*). HES proteins are transcription factors that are key mediators of the Notch signaling pathway and gamma-secretase activity is crucial for the activation of this pathway. So, based on the RNA-Seq results we subsequently treated spinal cord injured larval sea lampreys with a novel gamma-secretase inhibitor (PF-3804014). This treatment also reduced the expression of *HESB* in the brainstem and significantly enhanced the regeneration of individually identifiable descending neurons after a complete spinal cord injury. Our results show that gamma-secretase could be a novel target to promote axon regeneration after nervous system injuries.

## Introduction

In mammals, including humans, spinal cord injury (SCI) is a devastating event that can lead to permanent disability. The death of neurons that are not replaced after the injury and lack of axon regeneration of axotomized neurons are two of the main causes of permanent neurological deficits after SCI. In striking contrast to mammals, lampreys, as other basal vertebrates, are able to recover locomotion spontaneously after a complete SCI (Rodicio and Barreiro-Iglesias, 2012; Romaus-Sanjurjo et al., 2018b). During recovery from a complete SCI, lampreys are able to regenerate approximately 50% of their brainstem descending neurons and this partial axonal regeneration is needed for recovery (Davis and McClellan, 1994; Jacobs et al., 1997; Cornide-Petronio et al., 2011; Parker, 2017). This offers an interesting vertebrate model to find new signaling pathways responsible for neuronal survival and axon regeneration in descending neurons after SCI.

In a recent study, we showed that endogenous GABA promotes axon regeneration in descending neurons of lampreys after a complete SCI, and that GABOB (a GABA analog) and baclofen (a GABAB receptor agonist) treatments further promoted axon regeneration (Romaus-Sanjurjo et al., 2018a, 2019). GABA and baclofen treatments also inhibited caspase activation in giant individually identifiable descending neurons following the complete SCI (Romaus-Sanjurjo et al., 2018a). Here, we repeated the GABA and baclofen treatments and carried out 2 independent Illumina RNA sequencing (RNA-Seq) studies in the brainstems of control non-treated animals and treated animals. The aim of these RNA-Seq studies was to obtain gene expression data that could reveal secondary pathways modulated by GABAergic signaling in injured neurons and identify new signaling pathways that could be involved in the control of neuronal survival and axon regeneration in lampreys.

As shown below, results from these RNA-Seq experiments revealed changes in the expression of a *HES* gene. HES proteins are key mediators of the Notch signaling pathway (Engler et al., 2018). In the developing central nervous system, Notch signaling is mainly known for its role in neurogenesis and neuronal differentiation (see Imayoshi and Kageyama, 2011; Engler et al., 2018; Sagner et al., 2018), a feature that is conserved in developing lampreys (Lara-Ramirez et al., 2019). But, interestingly, a recent study showed that Notch signaling also inhibits axon regeneration in worms (El Bejjani and Hammarlund, 2012; Po et al., 2012). Whether this is also the case in vertebrates was not known. Here, we provide evidence showing that the pharmacological inhibition of gamma-secretase promotes axon regeneration after a complete SCI in lampreys.

## Materials and Methods

### Animals, SCI and Drug Treatments

All experiments involving animals were approved by the Bioethics Committee at the University of Santiago de Compostela and the *Conselleriìa do Medio Rural e do Mar* of the *Xunta de Galicia* (license reference JLPV/IId; Spain), and were performed in accordance with European Union and Spanish guidelines on animal care and experimentation. Animals were deeply anesthetized with 0.1% MS-222 (Sigma, St Louis, MO) in lamprey Ringer solution before all experimental procedures.

Mature and developmentally stable larval sea lampreys, *Petromyzon marinus* L. (*n* = 149; between 100 and 120 mm in body length, 5–7 years of age), were used in this study. Larval lampreys were collected from the river Ulla (Galicia, north-western Spain), with permission from the *Xunta de Galicia*, and maintained in aerated freshwater aquaria at 15°C with a bed of river sediment until their use in experimental procedures. Larval lampreys were randomly distributed between the different experimental groups.

**FIGURE 1:**
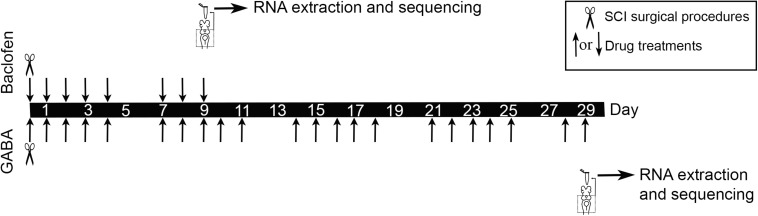
Experimental design for the RNA-Seq experiments.

SCI surgical procedures and drug treatments with GABA and baclofen were performed as previously described (Romaus-Sanjurjo et al., 2018a; Figure 1). Briefly, the rostral spinal cord was exposed from the dorsal midline at the level of the 5th gill. A complete spinal cord transection was performed with Castroviejo scissors and the spinal cord cut ends were visualized under the stereomicroscope. After SCI, animals were returned to individual freshwater tanks. Each injured animal was examined 24 h after surgery to confirm that there was no movement caudal to the lesion site. The animals were allowed to recover in individual freshwater tanks at 19.5°C. GABA and baclofen were applied in the water where the animals were left after the SCI surgical procedures (GABA at a concentration of 500 μM and baclofen at a concentration of 125 μM). GABA treated animals and their controls were processed for RNA extraction 29 days post-lesion (dpl) [when spontaneous axon regrowth begins to predominate Zhang et al. (2005); Figure 1]. Baclofen treated animals and their controls were processed for RNA extraction 9 days post-lesion [at a time in which caspase activation begins to be detected in injured neurons (Barreiro-Iglesias and Shifman, 2012, 2015) and axon retraction predominates Zhang et al., 2005; Figure 1].

PF-3804014 [a novel gamma-secretase inhibitor (Lanz et al., 2010) that has been used in recent studies to inhibit Notch signaling (e.g., Samon et al., 2012; Wu et al., 2017); Tocris Bioscience, United Kingdom] treatment after a complete SCI (see previous paragraph) was done as previously described for taurine treatments (Sobrido-Cameán et al., 2019). In these experiments, PF-3804014 was dissolved in lamprey Ringer solution (137 mM NaCl, 2.9 mM KCl, 2.1 mM CaCl2, 2 mM HEPES; pH 7.4) at a concentration of 5 mM, soaked in a small piece of Gelfoam (Pfizer; New York, NY) and placed on top of the site of injury at the time of spinal cord transection. Control animals went through the same surgical procedures but were only treated with Gelfoam soaked in lamprey Ringer. Axon regeneration was assessed 11 weeks post-lesion (wpl) in control (*n* = 16) and PF-3804014 (*n* = 21) treated animals using Neurobiotin (Vector, Burlingame, CA) as a retrograde tracer as previously described (Sobrido-Cameán et al., 2019). Briefly, at 11 wpl a second complete spinal cord transection was performed 5 mm below the site of the original transection and Neurobiotin (Vector) was applied in the rostral end of the transected spinal cord with the aid of a minute pin (#000). The animals were allowed to recover for 1 week to allow transport of the tracer from the application point to the neuronal soma of descending neurons. Since the original SCI was a complete spinal cord transection, only neurons whose axons regenerated at least 5 mm below the site of injury were labeled by the tracer. The presence of Neurobiotin in whole-mounted brains/spinal cords was detected using Avidin-D-FITC conjugated (Vector; 1:500).

### Imaging and Quantifications

A spectral confocal microscope (model TCS-SP2; Leica, Wetzlar, Germany) was used to acquire images of the whole-mounted brains/spinal cords. The identity of regenerated (Neurobiotin labeled) and non-regenerated identifiable descending neurons was determined for each brain. Giant descending neurons are always identifiable by their know location and big size (Jacobs et al., 1997) regardless of the presence of the tracer. Then, we calculated the percentage of regenerated identifiable neurons per animal and the total number of regenerated and non-regenerated neurons for all animals in the control and treated groups.

**FIGURE 2:**
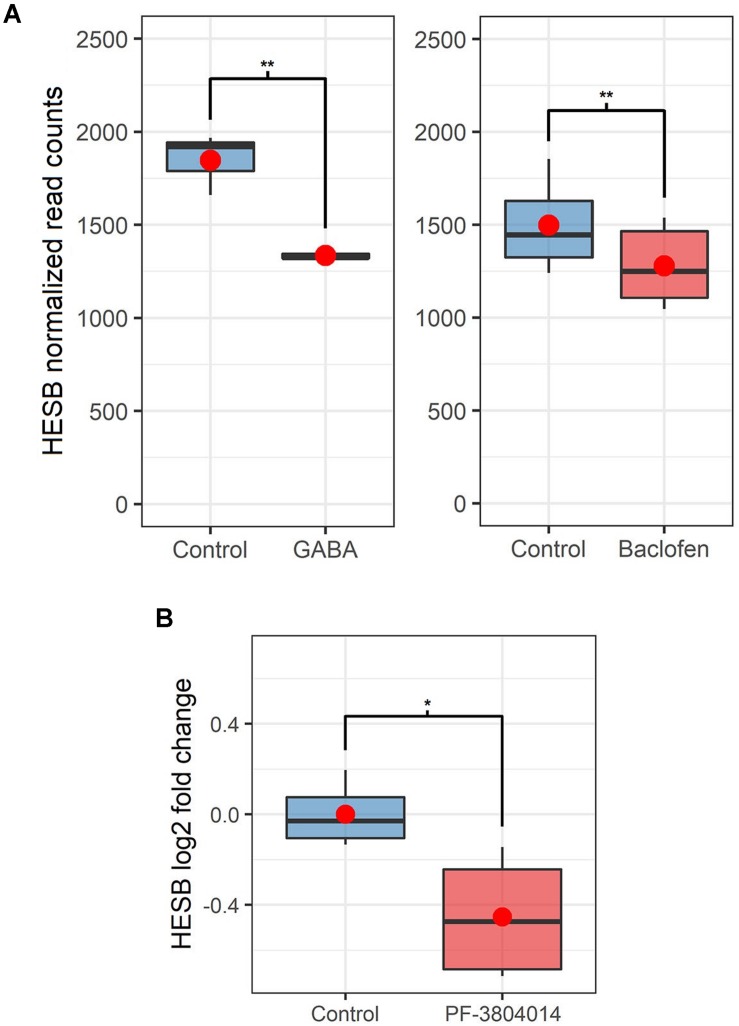
Changes in *HESB* expression in the brainstem of injured animals after drug treatments. **(A)** Changes in the expression of *HESB* in the brainstem of injured animals after GABA or baclofen treatments (RNA-Seq). *HESB* gene expression is represented as read counts normalized by DESeq’s median of ratios, and ** represents that the differences between the two groups are significant with false discovery rate corrected *p*-values between 0.01 and 0.001. Note that results are not comparable between the 2 RNA-Seq experiments. **(B)** Change in the expression of *HESB* in the brainstem of 1 wpl animals after the PF-3804014 treatment (qPCR). *HESB* expression is represented as log2 fold change in comparison to the mean of the controls, and * represents that the differences between the two groups are significant with *p*-value between 0.05 and 0.01. For both A and B, boxplots show the median, 25th and 75th percentiles, red dots represent the mean and whiskers extend to the most extreme data point which is no more than 1.5 times the length of the box away from the box.

For axon quantification, we scanned the rostral spinal cord (starting at the obex) using a 20x objective. Then, we traced a horizontal line through the middle of the spinal cord confocal stack (Figure 2A) using the Fiji software and manually quantified the number of axons crossing this line. To avoid missing overlapping axons we counted through the stack of optical sections using the counting plugin in Fiji. Only the axons that regenerate through the site of injury (ascending or descending) are labeled with the tracer. Note that with this preparation, we can only quantify the coarsest axons; therefore, it is likely that more axons actually regenerated both in control and treated animals. The experimenter was blinded during all quantifications.

### Total RNA Isolation

After the GABA or baclofen treatment periods (29 days in the GABA experiment and 9 days in the baclofen experiment) the brainstems of control and treated larvae were dissected out and immediately put in RNAlater (Ambion Inc., TX, United States) (Figure 1). RNA extraction was performed using the RNeasy mini kit (Qiagen, Germany) with DNase treatment following the manufacturer’s instructions. Isolated RNAs were eluted in nuclease free water. RNA quality and quantity were evaluated in a Bioanalyzer (Bonsai Technologies, Spain) and in a NanoDrop^®^ ND-1000 spectrophotometer (NanoDrop^®^ Technologies Inc., DE, United States). RNAs were stored at −80°C until use for RNA-Seq.

### RNA Sequencing

In the GABA experiment, 3 GABA treated and 3 control samples (each sample containing the RNA from 4 brainstems of 4 different animals; *n* = 24 in total) were sent to the Wellcome Trust Centre for Human Genetics (Oxford, United Kingdom) for library preparation and sequencing. RNA quality was checked again in a Bioanalyzer (Bonsai Technologies). All samples had RNA integrity number (RIN) of >8 (RIN measures how degraded the RNA is on a 0–10 scale, 10 being the least degraded). mRNA sequencing libraries were prepared from poly dT selected mRNA using the Illumina TrueSeq Stranded mRNA Library Preparation kit according to the manufacturer’s protocol. The average insert size of the libraries was approximately 300 bp. A different barcode was used for each sample to enable demultiplexing of the sequencing data. Sequencing was conducted on an Illumina HiSeq 4000 as 150 bp paired-end reads, yielding 23.2 to 27.8 million reads per sample.

In the baclofen experiment, 5 baclofen treated and 5 control samples (each sample containing the RNA of 8 brainstems of 8 different animals; *n* = 80 animals in total) were sent to Edinburgh Genomics (Edinburg, United Kingdom) for library preparation and sequencing. RNA quality was checked again in a Bioanalyzer (Bonsai Technologies). All samples had RNA integrity number (RIN) of >7.5. mRNA sequencing libraries were prepared from poly dT selected mRNA using the Illumina TrueSeq Stranded mRNA Library Preparation kit according to the manufacturer’s protocol. The average insert size of the libraries was approximately 300 bp. A different barcode was used for each sample to enable demultiplexing of the sequencing data. Sequencing was conducted on an Illumina NovaSeq S1 flowcell as 100 bp paired-end reads, yielding 26.8 to 37.6 million reads per sample.

### Quality Validation and Read Mapping

Between 23.2 and 37.6 million paired-end reads per sample were obtained (individual figures are shown in Supplementary Table S1). Sequencing quality was evaluated using FastQC. The quality of the baclofen experiment was significantly better than that of the GABA experiment (Supplementary Figure S1), non-etheless the sequencing output of both experiments showed average base qualities above 30 (reliability of the base call, the error probability of a quality of 30 is 0.001), except for the very last bases of the right reads of the GABA experiment (bases 141–150). Raw paired-end FASTQ files were quality filtered using Fastp (Chen et al., 2018) to remove leading or trailing bases with Phred quality scores <15 (‘-q 15’), reads with length <30 base pairs (‘-l 30’), polyG tails (‘–trim_poly_g’) and trim adaptor sequences (detected by per-read overlap, default). Significantly more reads were trimmed from the GABA experiment, congruent with the sequence quality assessment. The resulting trimmed FASTQ files were aligned against the latest lamprey genome assembly (Pmar_germline 1.0) (Smith et al., 2018) using STAR (Dobin et al., 2013) in 2-pass mapping mode (–twopassMode Basic) with the following parameters: 10 maximum multiple alignments allowed per read (–outFilterMultimapNmax 10), 10 maximum mismatches per read pair (–outFilterMismatchNmax 10), minimum and maximum intron lengths of 20 and 1 M bp, respectively (–alignIntronMin 20; –alignIntronMax 1000000), maximum distance of 1 Mb between mate reads (–alignMatesGapMax 1000000) and detection of chimeric sequences with a minimum mapped length of 20 bp (–chimSegmentMin 20; –chimScoreMin 1). The percentage of unique mapped reads ranged between 60.2 to 73.7%, with multimapping reads representing 9.3 to 26.1% and unaligned reads 13.1 to 18.1% (Supplementary Table S1).

### Transcriptome Reconstruction and Gene Expression

StringTie (Pertea et al., 2015) with default parameters (fr-firststrand, ‘–rf’) was used to construct the lamprey transcriptome from our samples using the genome alignment files. The resulting genome-guided transcriptome has 110,157 transcripts distributed in 57,134 genes. Transcript length ranged from 80 to 73,302 bp, with an average of 2314 bp, N50 of 5,304 bp and N90 of 987 bp. FeatureCounts (Liao et al., 2014) was used to quantify gene expression at the gene level with the following parameters: fragments (paired-end reads) were counted instead of individual reads (‘-p’), only fragments with both ends mapped were considered (‘-B’), chimeric fragments were not considered (‘-C’), and strand specified as reversely stranded (‘-s 2’). Statistical analyses related to differential expression were performed using R v.3.5.2^1^. The output matrix was input into the R/Bioconductor package DESeq2 (Love et al., 2014).

The Benjamini-Hochberg false discovery rate (FDR) was applied, and transcripts with corrected *P*-values < 0.05 were considered differentially expressed genes. Heatmaps were drawn using the R package ‘gplots’ v.3.01.1^2^. Pathway analyses were performed using Reactome (Fabregat et al., 2018), converting all non-human gene identifiers to their human equivalents.

### RNA-Seq Data Records

Raw FASTQ files for all samples were uploaded to the NCBI Sequence Read Archive (SRA), and are publicly available under BioProject accession numbers PRJNA561093 (GABA) and PRJNA561123 (Baclofen). The constructed transcriptome is available in the NCBI Transcriptome Shotgun Assembly (TSA) database under accession GHVE00000000. Read counts for each transcript were deposited in NCBI Gene Expression Omnibus (GEO) and are available under accession number GSE137860.

### qPCR Experiments After PF-3804014 Treatment

4 brainstems (*n* = 4 animals) from control 1 wpl vehicle treated animals and 4 brainstems (*n* = 4 animals) from 1 wpl PF-3804014 treated animals were processed for qPCR analyses of *HESB* (GenBank accession number: MN833804) expression. Specific primers for targeted genes were designed using Primer3: *HESB*-forward AAAGCAAAGACGGGACAGGA, *HESB*-reverse GATGTCCGCCTTCTCCAGTT, *GAPDH*-forward CAGATTCGTCCAGCTCCGTT, *GAPDH*-reverse CCATTGTGGAGCGGATGTCA. qPCR was performed on a Stratagene Mx3005P thermocycler (Agilent Technologies) using Brilliant III Ultra-Fast SYBR Green QPCR Master Mix in a final volume of 12.5 μL following the manufacturer’s protocol with 1 μL of cDNA per reaction. Primer concentration was 300 nM and each sample was run in duplicate. The cycling parameters were: 50°C for 2 min, 95°C for 10 min, followed by 40 cycles of amplification at 95°C for 15 s and 60°C for 1 min. After amplification, a dissociation step was performed raising the temperature from 65 to 95°C to create a melting curve and ensure the presence of a single amplification product. qPCR data were obtained by the MxPro software (Agilent Technologies) and quantification cycle values (Cq) calculated for each replicate and then averaged to obtain the final Cq value. *HESB* Cq values were normalized using the reference gene (*GAPDH*), and fold changes were calculated for each sample using the average of the controls as reference.

### Statistical Analyses

Statistical analysis was carried out using Prism 6 (GraphPad software, La Jolla, CA). Data were presented as mean ± SEM. Normality of the data was determined by D’Agostino–Pearson omnibus test and all data passed the normality test. A Student’s *t*-test was used to determine significant differences in the qPCR experiment and between control and PF-3804014 treated animals (percentage of regenerated neurons per animal and number of regenerated axons per animal). A Fisher’s exact test was used to determine significant differences between control and PF-3804014 treated animals (total number of regenerated neurons).

## Results

### RNA-Seq Differential Expression Analyses

In previous work, we showed that both GABA and baclofen promote axon regeneration after a complete SCI in lampreys (Romaus-Sanjurjo et al., 2018a). Here, we aimed to identify new genes involved in axon regeneration in lampreys that might be regulated by GABAergic signaling. For this we carried out 2 independent RNA-Seq studies in the brainstems of injured animals after GABA or baclofen treatments. 23 differentially expressed genes were detected between GABA-treated and control samples, and 82 differentially expressed genes between baclofen-treated and control samples (Supplementary File 1). At 29 dpl, treatment with GABA induced mostly the downregulation of genes compared to control animals (Supplementary File 1; 7 up-regulated and 16 down-regulated genes). The opposite trend was seen at 9 dpl with baclofen, which induced the upregulation of a higher number of genes (Supplementary File 1; 63 upregulated and 18 downregulated genes). This included changes in the expression of genes known to promote axon regeneration, like the transcription factor KLF7 (increased expression after baclofen treatment; see discussion).

A HES transcription factor [annotated as *HESB* in another lamprey species *Lampetra planeri* (Lara-Ramirez et al., 2019)] was the most significantly downregulated gene in response to GABA (logFC = −0.47; Figure 2A, and Supplementary File 1); and it was also one of the most significantly down-regulated genes in response to baclofen (logFC = -0.44; Figure 2; Supplementary File 1). The sea lamprey *HESB* transcript sequence was deposited in GenBank under accession number MN833804. HES proteins are key transcription factors in the Notch signaling pathway (Ohtsuka et al., 1999). Concordantly, Reactome pathway analysis revealed a significant enrichment of many pathways relating to Notch signaling (FDR *p*-value < 0.05) amongst differentially expressed genes after both treatments (see Supplementary File 2). This suggests that Notch signaling might play a crucial role in axon regeneration in lampreys.

### Pharmacological Inhibition of Gamma-Secretase Promotes Axon Regeneration After a Complete SCI

**FIGURE 3:**
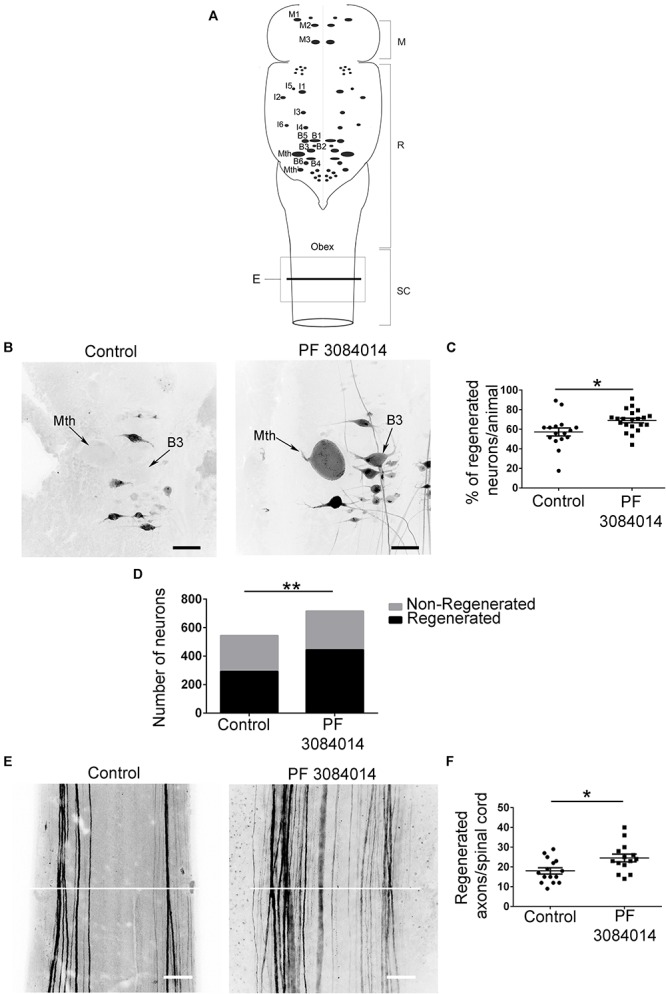
PF-3804014 promotes axon regeneration after a complete SCI. **(A)** Schematic drawing of a dorsal view of the sea lamprey brainstem showing the location of giant individually identifiable descending neurons (modified from Sobrido-Cameán and Barreiro-Iglesias, 2018). The box marked “E” indicates the spinal cord region shown in Panel E. The line used to count the number of regenerated axons in the spinal cord is also indicated. M, mesencephalon; R, rhombencephalon; SC, spinal cord. **(B)** Photomicrographs of dorsal views of whole-mounted brains showing regenerated identifiable descending neurons, as identified by retrograde tracer labeling, in control and PF-3804014 treated animals. Note the increased number of labeled (regenerated) identifiable neurons in PF-3804014 treated animals. **(C)** Graph showing significant changes (asterisk) in the percentage of regenerated identifiable descending neurons per animal (controls: *n* = 16 animals; PF-3804014 treated: *n* = 21 animals) after the PF-3804014 treatment (control: 57,22 ± 4.132%; PF-3804014: 68.95 ± 2.354%). **(D)** Graph showing significant changes (asterisks) in the total number of regenerated neurons after the PF-3804014 treatment (control: 292 neurons regenerated, 252 non-regenerated; PF-3804014: 444 neurons regenerated, 272 non-regenerated). **(E)** Photomicrographs of whole-mounted spinal cords showing regenerated axons, as identified by tracer labeling, in control and PF-3804014 treated animals. Note the increased number of regenerated axons in PF-3804014 treated animals. The line used to count the number of regenerated axons in the spinal cord is also indicated in white. **(F)** Graph showing significant changes (asterisk) in the number of regenerated axons per spinal cord after the PF-3804014 treatment (control: 18 ± 1.588 axons, *n* = 15 animals; PF-3804014: 24.57 ± 1.955 axons, *n* = 14 animals). Rostral is up and scale bars correspond to 100 μm.

Gamma-secretase activity is crucial for the release of the Notch intracellular domain (NICD) to activate the transcription of target genes like the HES family of transcription factors (see Fischer and Gessler, 2007). So, based on the RNA-Seq results we decided to treat larval sea lampreys after a complete spinal cord transection with the novel gamma-secretase inhibitor PF-3804014. First, qPCR experiments showed that the PF-3804014 treatment also caused a significant decrease in *HESB* expression in the brainstem of spinal cord injured animals (1 wpl) as compared to vehicle treated controls (logFC = −0.45; Student’s *t*-test, *p* = 0.0307; Figure 2B). At 11 wpl, the PF-3804014 treatment significantly promoted axon regeneration of individually identifiable descending neurons (Figure 3A; the M1-3, I1-6, B1-B6, Mth, and Mth’ descending neurons) of the sea lamprey (Figures 3B–D); both when looking at the percentage of regenerated identifiable descending neurons per animal (Student’s *t*-test, *p* = 0.0132; Figures 3B,C) or at the total number of regenerated identifiable neurons (Fisher’s exact test, *p* = 0.0032; Figures 3B,D). Moreover, the acute PF-3804014 treatment significantly increased the number of regenerated axons (ascending or descending) in the spinal cord following a complete SCI (Student’s *t*-test, *p* = 0.0134; Figures 3E,F). These results show that inhibiting gamma-secretase activity acutely after a complete spinal cord transection enhances true axon regeneration in sea lamprey neurons.

## Discussion

Our 2 RNA-Seq studies in the brainstem of spinal cord injured larval sea lampreys revealed a significant downregulation of a HES transcription factor (*HESB*) and a significant enrichment of the Notch signaling pathway after GABA and baclofen treatments. The fact that this was detected in 2 independent RNA-Seq studies carried out in different sequencing platforms, using 2 different GABAergic drugs and at 2 different time points after injury provided strong confidence on the possible importance of this signaling pathway during regeneration. Gamma-secretase activity is crucial for the release of NICD to activate the transcription of target genes like the HES family of transcription factors (see Fischer and Gessler, 2007). Treatments with the gamma-secretase inhibitor PF-3804014 also caused a significant decrease in the expression of *HESB* in the brainstem and a significant increase in axon regeneration after a complete SCI. Specifically, this treatment led to a significant increase in the regeneration of individually identifiable descending neurons of the lamprey brainstem. This is in agreement with our previous results showing improved axon regeneration after baclofen or GABOB treatments after SCI in lampreys (Romaus-Sanjurjo et al., 2018a). So, the Notch signaling pathway appears to be of crucial importance in the regulation of axon regrowth after SCI in lampreys. Our results also suggest that GABA signaling promotes axon regeneration, at least partially, by inhibiting the Notch signaling pathway.

A previous study in *Caenorhabditis elegans* revealed that Notch signaling inhibits axon regeneration in identifiable neurons of this nematode (El Bejjani and Hammarlund, 2012). Importantly, our findings extend these results to vertebrates and the SCI situation. As in lampreys, treatments with a gamma-secretase inhibitor (DAPT in their case) improved axon regeneration in worms (El Bejjani and Hammarlund, 2012). Interestingly, gamma-secretase activity had to be inhibited right after injury in worms to improve regeneration (El Bejjani and Hammarlund, 2012), which coincides with our results using also early pharmacological inhibition in lampreys. In *C. elegans*, genetic manipulations showed that Notch signaling acts autonomously in the injured neuron to prevent growth cone formation (El Bejjani and Hammarlund, 2012). Our current data in lampreys analyzing whole brainstems (which include descending neurons but also other types of neurons and glial cells) in the RNA-Seq/qPCR experiments and pharmacological inhibition (present results) does not allow us to differentiate between direct or indirect effects of this signaling pathway in the regeneration of descending neurons. In future work it would be important to study if Notch signaling acts directly on lamprey descending neurons during regeneration or if the positive effects of gamma-secretase inhibition come through an indirect mechanism.

Notch1 expression increases in the rodent spinal cord after SCI (Yamamoto et al., 2001; Chen et al., 2005), and treating human induced pluripotent stem cell-derived neural stem/progenitor cells with a gamma-secretase inhibitor promoted axon growth from these cells after transplantation to the injured spinal cord of mice (Okubo et al., 2018). So, putting together previous data in rodents and our results in lampreys we propose that gamma-secretase could be an interesting target to promote axon regeneration after SCI in mammalian models. Moreover, gamma-secretase could also serve as a target to promote other regenerative aspects, for example gamma-secretase inhibition promotes stem cell proliferation and generation of new motor neurons after SCI in zebrafish (Dias et al., 2012). Future work should test the effects of gamma-secretase inhibition in preclinical rodent models of SCI to evaluate both positive and negative outcomes of this treatment.

Finally, it is important to mention that our 2 RNA-Seq studies revealed significant changes in the expression of other genes (see Supplementary File 1), which provides a resource for future studies on the role of these genes during regeneration. A comparison between the GABA and baclofen RNA-Seq experiments reveals differences in the changes of gene expression (see Supplementary File 1). This might be related to the different time points of analysis (29 or 9 dpl) and due to a potential influence of GABAA receptors during GABA treatment. Changes in gene expression after SCI in lampreys have been also observed even at later time points when looking at the expression of specific genes like with the *HDAC1* gene (Chen et al., 2016); therefore, new RNA-Seq studies at different time points might reveal more genes involved in the positive effects of GABA signaling. Regarding a possible contribution of GABAA receptors, we have recently shown that a muscimol (a GABAA agonist) treatment inhibits caspase activation in identifiable descending neurons of lampreys after a complete SCI (Sobrido-Cameán et al., 2018), which supports the possibility of an influence of GABAA receptors during the GABA treatment.

As an example of other genes, the baclofen treatment caused a significant increase in the expression of 2 members of the Krüppel-like family of transcription factors (KLFs): *KLF7* and *KLF10*. Changes in the expression of different KLFs (including *KLF7* and *KLF10*) were previously detected over the course of recovery in an RNA-Seq study after SCI in lampreys (Herman et al., 2018). Previous work in mammalian models has revealed a differential implication of different KLFs in the control of axon regeneration after different types of nervous system injuries Moore et al., 2009 (for reviews see Moore and Goldberg, 2011; Moore et al., 2011). KLF7 promotes axon regeneration in mammals (Blackmore et al., 2012; Wang et al., 2016), which coincides well with our results revealing increased expression of KLF7 after baclofen treatment in lampreys with a complete SCI. As far as we are aware, the implication of KLF10 in axon regeneration has not yet been elucidated. Our results open the way to study the role of these or other genes in both neuronal survival and axon regeneration after SCI.

## Data Availability Statement

Raw FASTQ files for all samples were uploaded to the NCBI Sequence Read Archive (SRA), and are publicly available under BioProject accession numbers PRJNA561093 (GABA) and PRJNA561123 (Baclofen). The constructed transcriptome is available in the NCBI Transcriptome Shotgun Assembly (TSA) database under accession GHVE00000000. Read counts for each transcript were deposited in NCBI Gene Expression Omnibus (GEO) and are available under accession number GSE137860.

## Ethics Statement

The animal study was reviewed and approved by the Bioethics Committee of the University of Santiago de Compostela Santiago de Compostela, A Coruña, Spain.

## Author Contributions

DS-C and DR-S performed the SCI surgeries, drug treatments and brainstem dissections. VP-C performed the RNA extractions. DR performed the bioinformatic analyses. LS, MR, and AB-I supervised the study. MR and AB-I conceived the study. DR and AB-I wrote the manuscript. All authors have approved the final manuscript.

## Conflict of Interest

The authors declare that the research was conducted in the absence of any commercial or financial relationships that could be construed as a potential conflict of interest.

## Footnotes

1 http://www.R-project.org/

2 https://cran.r-project.org/web/packages/gplots/index.html
