# Supplementary material for: Inhibition of Gamma-Secretase Promotes Axon Regeneration After a Complete Spinal Cord Injury
Source: Front Cell Dev Biol. 2020 Mar 20;8:173. doi: 10.3389/fcell.2020.00173 (PMC7100381; doi:10.3389/fcell.2020.00173)

**Supplementary Figure 1. Quality assessment of raw paired-end FASTQ files.** Box and whisker plots show the phred quality distribution per base for the left and right paired-end FASTQ files of one representative sample of the GABA experiment (sequenced on a HiSeq as 150 bp PE) and one representative sample of the Baclofen experiment (sequenced on a NovaSeq as 100 bp PE).

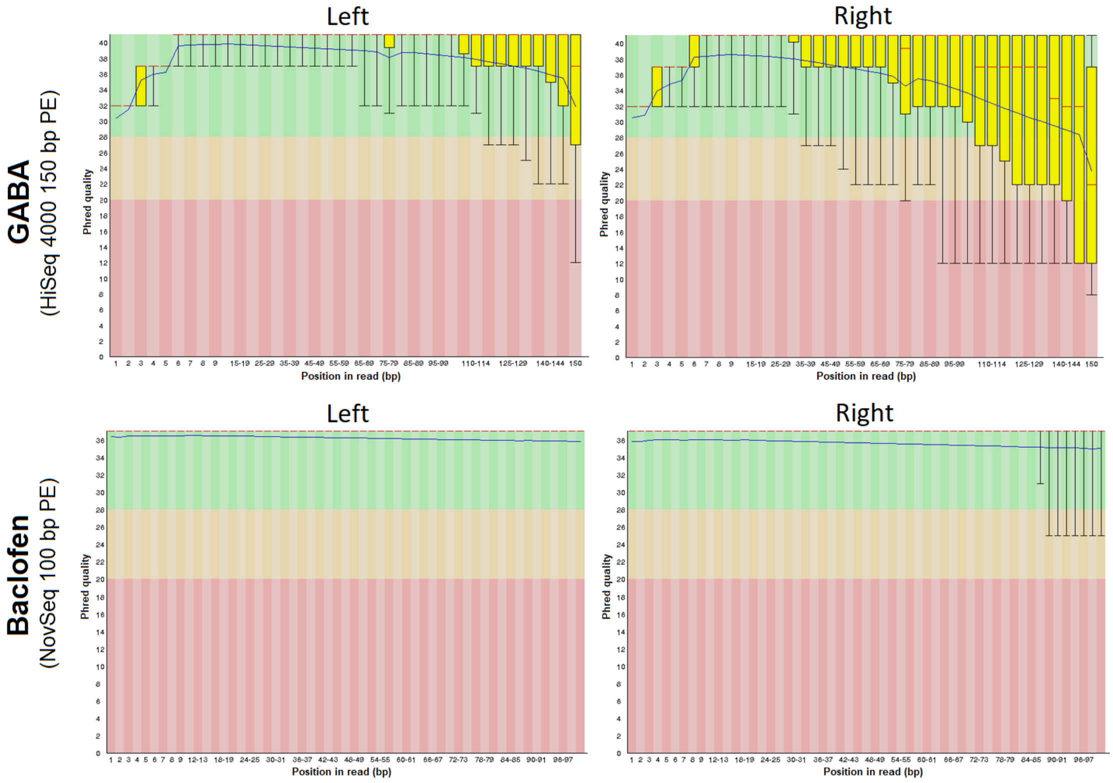

Supplement: Supplementary file 1 [file Image_1.pdf]
