# Supplementary material for: Inhibition of Gamma-Secretase Promotes Axon Regeneration After a Complete Spinal Cord Injury
Source: Front Cell Dev Biol. 2020 Mar 20;8:173. doi: 10.3389/fcell.2020.00173 (PMC7100381; doi:10.3389/fcell.2020.00173)
Supplement: Supplementary file 2 [file Table_1.pdf]

Supplementary Table 1. Read quality, trimming and alignment statistics.

Sample Name	Sequencer	Read length (bp)	Raw read-pairs	Trimmed read-pairs	Uniquely mapped reads (%)	Multimapping reads (%)	Unaligned reads (%)
Gaba1	HiSeq 4000	2 x 150	26076936	23109121	73.69	10.16	16.15
Gaba2	HiSeq 4000	2 x 150	26672802	22272653	72.21	9.76	18.03
Gaba3	HiSeq 4000	2 x 150	27701871	21614762	72.84	9.95	17.21
Gaba_control1	HiSeq 4000	2 x 150	27321787	22850845	72.2	9.92	17.88
Gaba_control2	HiSeq 4000	2 x 150	24466255	20360622	71.85	10.07	18.08
Gaba_control3	HiSeq 4000	2 x 150	23265385	19315039	73.26	9.33	17.41
9dpi_Baclofen1	NovaSeq S1	2 x 100	27221968	26695151	69.87	15.22	14.91
9dpi_Baclofen2	NovaSeq S1	2 x 100	33794539	33137166	60.26	26.02	13.72
9dpi_Baclofen3	NovaSeq S1	2 x 100	31635757	31140362	70.35	16.55	13.1
9dpi_Baclofen4	NovaSeq S1	2 x 100	37581230	36818848	69.25	15.57	15.18
9dpi_Baclofen5	NovaSeq S1	2 x 100	37024797	36373423	70.12	15.1	14.78
Baclofen_control1	NovaSeq S1	2 x 100	36911284	36305040	66.99	18.74	14.27
Baclofen_control2	NovaSeq S1	2 x 100	32095086	31500785	68.18	18.53	13.29
Baclofen_control3	NovaSeq S1	2 x 100	26842004	26396100	64.13	21.95	13.92
Baclofen_control4	NovaSeq S1	2 x 100	30719653	30191932	69.04	16.44	14.52
Baclofen_control5	NovaSeq S1	2 x 100	33187409	32643024	66.91	18.59	14.5
